# Spatiotemporal Variability of Asymmetric Daytime and Night-Time Warming and Its Effects on Vegetation in the Yellow River Basin from 1982 to 2015

**DOI:** 10.3390/s19081832

**Published:** 2019-04-17

**Authors:** Liqun Ma, Haoming Xia, Qingmin Meng

**Affiliations:** 1 The College of Environment and Planning, Key Laboratory of Geospatial Technology for Middle and Lower Yellow River Regions, Henan Collaborative Innovation Center of Urban-Rural Coordinated Development, Henan University, Kaifeng 475004, China; mayifan18@163.com; 2 Department of Geosciences, Mississippi State University, Starkville, MS 39762, USA; qmeng@geosci.msstate.edu

**Keywords:** Yellow River Basin, growing season, daytime and night-time warming, asymmetric warming, NDVI

## Abstract

Temperatures from 1982 to 2015 have exhibited an asymmetric warming pattern between day and night throughout the Yellow River Basin. The response to this asymmetric warming can be linked to vegetation growth as quantified by the NDVI (Normalized Difference Vegetation Index). In this study, the time series trends of the maximum temperature (*T_max_*) and the minimum temperature (*T_min_*) and their spatial patterns in the growing season (April–October) of the Yellow River Basin from 1982 to 2015 were analyzed. We evaluated how vegetation NDVI had responded to daytime and night-time warming, based on NDVI and meteorological parameters (precipitation and temperature) over the period 1982–2015. We found: (1) a persistent increase in the growing season *T_max_* and *T_min_* in 1982–2015 as confirmed by using the Mann–Kendall (M–K) non-parametric test method (*p* < 0.01), where the rate of increase of *T_min_* was 1.25 times that of *T_max_*, and thus the diurnal warming was asymmetric during 1982–2015; (2) the partial correlation between *T_max_* and NDVI was significantly positive only for cultivated plants, shrubs, and desert, which means daytime warming may increase arid and semi-arid vegetation’s growth and coverage, and cultivated plants’ growth and yield. The partial correlation between *T_min_* and NDVI of all vegetation types except broadleaf forest is very significant (*p* < 0.01) and, therefore, it has more impacts vegetation across the whole basin. This study demonstrates a methodogy for studying regional responses of vegetation to climate extremes under global climate change.

## 1. Introduction

As an important part of the global terrestrial ecosystem, vegetation is playing a major role in modulating regional and global climate change through biogeochemical and biophysical feedbacks [1]. Dynamic changes of vegetation and its response to climate change have become the critical issues in the study of global climate change [2,3]. Climate change is significantly affecting vegetation growth. Temperature is one of the most important factors that control vegetation growth and production [4]. The Intergovernmental Panel on Climate Change (IPCC) Assessment Report provided further study results that climate warming is asymmetric (IPCC 2013) [5]. The increasing rate of daily minimum temperatures (*T_min_*) is globally 1.4 times the daily maximum temperature (*T_max_*), which is known as asymmetric warming [6,7]. Increased temperature, particularly asymmetric climate warming would affect plant growth and above-ground biomas. Since most plants photosynthesize during the daytime, plant growth may be more sensitive to *T_max_* than *T_min_*; plant respiration occurs throughout the whole day and, therefore, both *T_max_* and *T_min_* influence respiration [7].

At present, many studies have showed that temperature rise was asymmetric globally, and vegetation has different responses to the rises of maximum temperature and minimum temperature, using NDVI (Normalized Difference Vegetation Index) as the response to temperature [7,8,9,10,11,12,13,14,15,16,17]. NDVI can serve as an excellent indicator for vegetation dynamics by representing green leaf biomass and productivity, and has been regarded and used as a powerful indicator of vegetation changes [18]. The study of the dynamic change of vegetation and its responses to climate warming is of great significance to understanding surface hydrothermal processes, carbon balance processes, and the prediction of dynamic changes in terrestrial ecosystems [19]. Therefore, it is necessary to study the influence of diurnal extreme temperatures on vegetation growth. 

The response and sensitivities of vegetation to diurnal asymmetry have been confirmed in some studies. For example, relevant researchers have found that the increasing *T_max_* is conducive to vegetation growth and carbon sink of vegetation in most frigid and temperate humid regions, but not the case in temperate arid and semi-arid regions [7,8,14,15,16,20]. However, the increasing *T_min_* during growing seasons can have the opposite effect on vegetation growth. At the same time, it was found that the increasing *T_min_* at night promoted alpine grasslands and meadows, and inhibited forest in the humid regions [15]. The carbon sequestration capacity of typical grassland ecosystems in the north is raised by the increasing temperature at night [12]. *T_min_* can promote the growth of grassland (as measured by NDVI) in Inner Mongolia [20]. The proportion of the area where *T_min_* has a significant positive influence on vegetation NDVI is more than that of *T_max_* in Xinjiang [13]. However, the partial correlation between night-time temperature increasing and NDVI in the Nansi Lake wetland (a humid region) was not significant during the study period [21]. 

Therefore, it is necessary to conduct an extensive study across a large region including multiple climate zones and diverse vegetation types to have a comprehensive understanding of how climate asymmetric warming can impact vegetation dynamics. The Yellow River Basin, which spans the arid, semi-arid and arid climate zones in the north of China, is sensitive to global climate change because it faces serious water deficit problems and is thus one of the ideal regions for examing the asymmetric warming’s effects on vegetation [22,23,24]. Although some studies pointed out that the differences in hydrothermal climate distribution across the Yellow River Basin could lead to different sensitivities of vegetation to diurnal asymmetric warming in different regions [25,26,27,28,29], there is limited understanding about the effect and influence of asymmetric warming on the vegetation dynamics across the Yellow River Basin. However, the special natural environment of the Yellow River Basin is also a good research area, which is used to verify the relationships between vegetation and hydrothermal climate change at the local scale [23,24]. Based on the GIMMS (Global Inventory Monitoring and Modelling System), NDVI3g data from 1982 to 2015, we investigated the time-series trends of daytime and night-time warming, and analyzed the asymmetric warming effects on vegetation across the Yellow River Basin, which is very significant for the ecological, environmental, and agricultural adaptation to climate changes. The findings in this study can provide deep understanding of the vegetation dynamics in the growing season in response to climate change and inform decision making for sustainable development of ecological systems over the Yellow River Basin. 

## 2. Materials and Methods

### 2.1. Data Sources and Processing

#### 2.1.1. Meteorological Data

**Figure 1:**
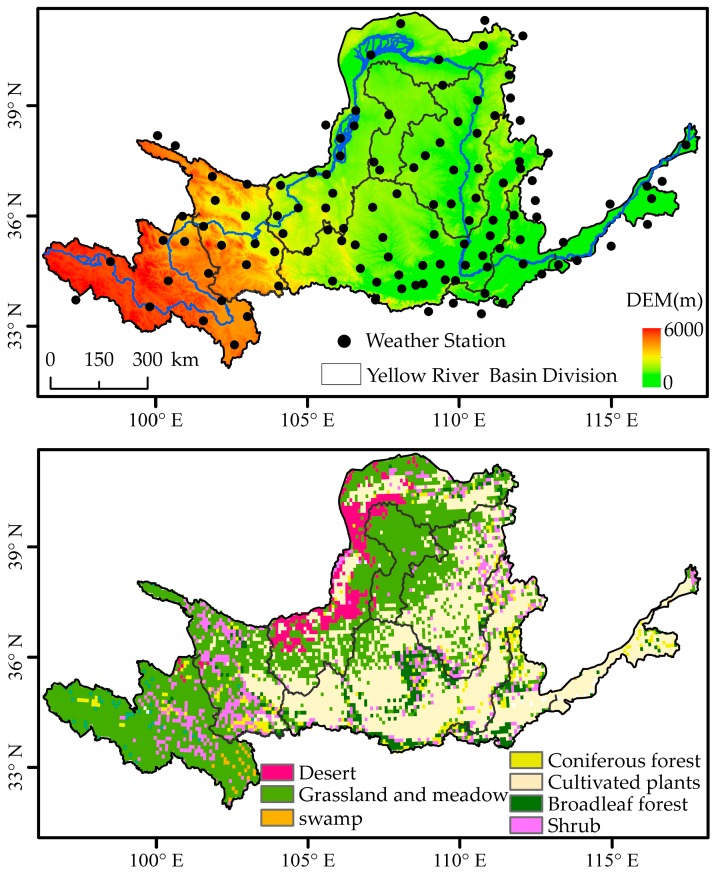
The altitude, weather stations, and vegetation types in the Yellow River Basin.

All the meteorological data of the study underwent strict correction and quality control, which were downloaded from the National Meteorological Information Center [30]. In order to identify structural changes or possible changing points in the data series, the RHtest V4 software was used to test a homogeneity, based on principles provided by Wang [25,31,32]. The criterion for inclusion of a meteorological station was that its recording time had to include at least 85% of the total 34-year period [25]. After all the meteorological data quality control and homogeneity assessments, the 34-year data (from 1 January 1982 to 31 December 2015) from 118 stations were used (Figure 1). 

The spatial distribution of temperature and precipitation was significant correlated with the altitude and terrain, therefore, the digital elevation model (DEM) was required as co-variate [33,34]. Based on the DEM provided by the National Aeronautics and Space Administration (NASA) Shuttle Radar Topographic Mission (SRTM) [35], we used the DEM as a co-variable and employs thin plate smoothing splines to interpolate temperature and precipitation values across the study area. Thin plate smoothing splines was chosen because it has been used in other global studies [36,37], performed well in comparative tests of multiple interpolation techniques [38], and because it is computationally efficient and easy to run. All the meteorological data (temperature and precipitation) were interpolated into a raster dataset of an 8 km spatial resolution. The *T_max_* and *T_min_* during the growing season (from April to October) were calculated for the same days each year, respectively.

#### 2.1.2. Normalized Difference Vegetation Index (NDVI)

The purpose of this study is to detect change of NDVI for a long time in the Yellow River Basin. The third-generation Normalized Difference Vegetation Index (NDVI3g) dataset published by NASA’s Global Inventory Modeling and Mapping Studie (GIMMS) group was the longest NDVI time series to date [39], compared to SPOT (Systèm Pour l’Observation de la Terre) and MODIS (Moderate Resolution Imaging Spectroradiometer) data. The GIMMS-NDVI3g dataset with a spatial resolution of 1/12 degree and a time interval of 15 days [36], which were powerful tool that assess the photosynthetic activities of vegetation and have been atmospheric radiation, radiation corrected and coordinate transformation. The half-month NDVI data were processed by the maximum synthesis method to synthesize monthly data [40].

The NDVI during the growing season (from April to October) was defined as the average monthly composite NDVI [4,15,41]. In sparsely and bare vegetated zones, there have interference of soil variation on spectral signal. Therefore, our study only considered the pixels, which have an average growing-season NDVI greater than 0.1 [15,16]. 

#### 2.1.3. Vegetation Data

This study utilized maps of China’s vegetation cover at a scale of 1:1,000,000 published by the Cold and Arid Regions Science Data Center, Chinese Academy of Sciences (CAS) [42]. The main vegetation types were reclassified as coniferous forest, broadleaf forest, shrub, cultivated plants, grassland and meadow, swamp, and desert (Figure 1). In order to match the spatial resolution of GIMMS NDVI3g data, the vegetation data was resampled a spatial resolution of 8 km using a majority filter method.

### 2.2. Methods

#### 2.2.1. Trend Analyses and Mann–Kendall (M–K) Test

We used the slope of linear regression to investigate the change of *T_max_* (and *T_min_*) across the Yellow River Basin from 1982 to 2015. In order to test the significance of the change trend, the Mann–Kendall non-parametric test method (M–K) was used to investigate the trend of the maximum temperature (*T_max_*) and the minimum temperature (*T_min_*) at regional and pixel scales [43,44,45]. The significance of the linear regression coefficient was assessed by t-test, where *p* < 0.05 indicates that the regression coefficient is significant, and *p* < 0.01 indicates that the regression coefficient is very significant. The spatiotemporal variabilities of the *T_max_* and *T_min_* in the Yellow River Basin were analyzed at the pixel level.

#### 2.2.2. Partial Correlation Analysis

The partial correlation analysis was applied to study the influence of the asymmetry of day and night warming on the NDVI by controlling the interferences of other variables [46]. In this study, the second-order partial correlation coefficient of NDVI and *T_max_* or *T_min_* is calculated pixel by pixel. In other words, the partial correlation coefficient of NDVI and *T_max_* is calculated by controlling *T_min_* and precipitation; the partial correlation between NDVI and *T_min_* is calculated by controlling *T_max_* and precipitation. The t-test was also used to test the significance of the partial correlation coefficient.

## 3. Results and Analysis

### 3.1. The Spatial and Temporal Patterns of Daytime and Night-Time Warming

**Figure 2:**
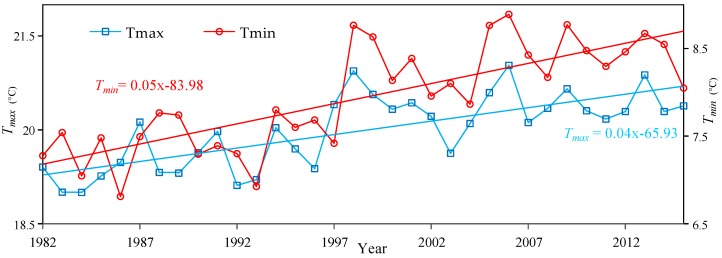
Variations of mean *T_max_* and *T_min_* during the growing seasons from 1982 to 2015 in the Yellow River Basin.

**Figure 3:**
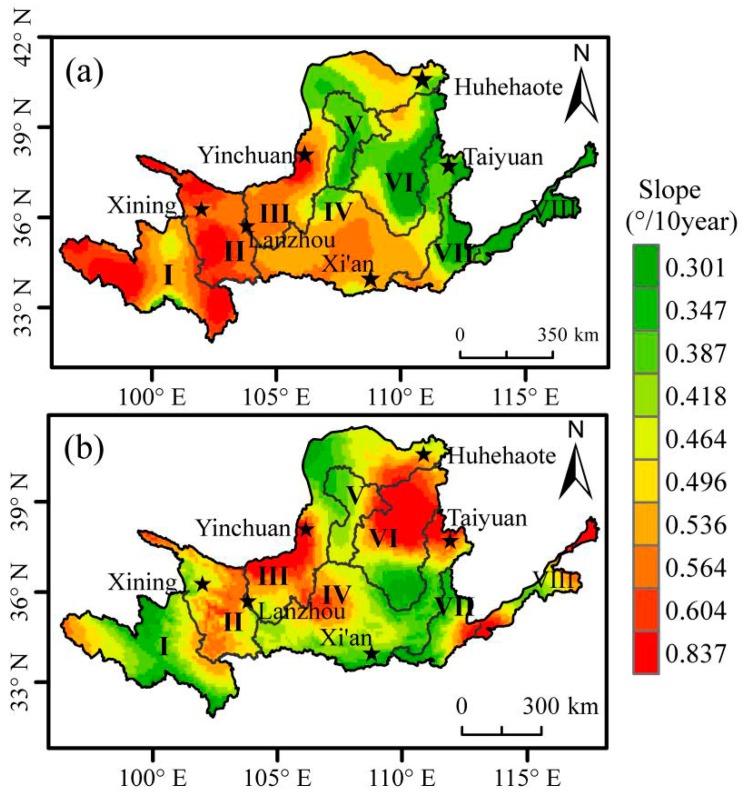
Air temperature trends in growing season across the Yellow River Basin over the period 1982–2015. (**a**) the slope of *T_max_*; (**b**) the slope of *T_min_*; (I: Longyangxia in the upper reaches; II: Longyangxia to Lanzhou; III: Lanzhou to Hekou town; IV: Longmen to Sanmenxia; V: Inflow zone; VI: Hekou town to Longmen area; VII: Longmen to Huayuankou; VIII: below Huayuankou in the lower reaches).

The trend of the *T_max_* and *T_min_* was calculated at the 95% confidence level (Figure 2 and Figure 3). Figure 2 shows the increasing trends of *T_max_* and *T_min_* during the 1982–2015 period. *T_min_* had the highest and most significant temperature increases (0.5 °C/10 years) (*p* < 0.05), and *T_max_* also showed a significant increasing trend (0.4 °C/10 years) (*p* < 0.05). *T_min_* increased by about 1.25 times (0.5/0.4) as much as *T_max_* over the Yellow River Basin during 1982 to 2015. 

Over the period 1982–2015, *T_max_* of the Yellow River Basin showed a significant increasing trend (Figure 3a) and about 97.9% of the study area showed a significant increasing trend (*p* < 0.05), among which about 90.2% showed a very significant increasing trend (*p* < 0.01). The regions with the fastest increasing trend were mainly distributed above Longyangxia in the upper reaches (I) of the Yellow River Basin and from Longyangxia to Lanzhou (II). The regions with the slowest increasing trend were distributed below Huayuankou in the lower reaches (VIII) of the Yellow River Basin (Figure 3a).

In 1982–2015, *T_min_* of the Yellow River Basin showed a significant increasing trend (*p* < 0.05), of which about 99.5% showed an extremely significant increasing trend (*p* < 0.01) (Figure 3b). The regions with the fastest increasing trend are mainly distributed from Lanzhou to Hekou town (III), and from Hekou town to the north of Longmen area (VI). The regions with the slowest increasing trend are distributed in the north of Longyangxia area and above (I), from Hekou town to the south of Longmen area, and from Longmen to the east of Sanmenxia area (IV) (Figure 3b).

### 3.2. Partial Correlation between NDVI and Daytime and Night-Time Warming

**Figure 4:**
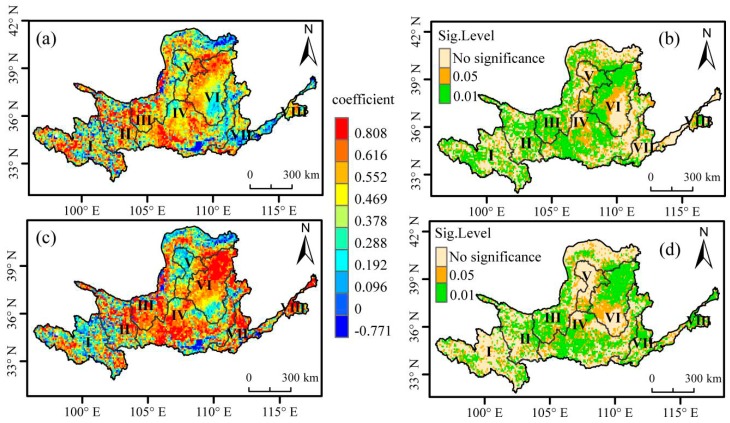
Spatial patterns of the correlations between Normalized Difference Vegetation Index (NDVI) and *T_max_* or *T_min_* during the growing season (April–October) in the Yellow River Basin, 1982–2015. (**a**) Mapping of the partial correlation coefficients between NDVI and *T_max_*, given that the corresponding *T_min_* and precipitation are controlled for in the calculation. (**b**) Spatial distribution of significance level of the partial correlation coefficients between NDVI and *T_max_*. (**c**) The partial correlation coefficients between NDVI and *T_min_* by controlling *T_max_* and precipitation. (**d**) Spatial distribution of partial correlation coefficients between NDVI and *T_min_*.(I: Longyangxia in the upper reaches; II: Longyangxia to Lanzhou; III: Lanzhou to Hekou town; IV: Longmen to Sanmenxia; V: Inflow zone; VI: Hekou town to Longmen area; VII: Longmen to Huayuankou; VIII: below Huayuankou in the lower reaches).

The second-order partial correlation analysis of NDVI and maximum temperature or minimum temperature during the growing season was carried out in the study area at a pixel level (Figure 4). As the effects of *T_min_* and precipitation in growing season were controlled in the partial correlation, about 87.1% of the study areas had positive correlation between NDVI and *T_max_*, of which 20.1% showed very significant positive correlation (*p* < 0.01) and 23.5% showed significant positive correlation (*p* < 0.05). Although there was a negative correlation between NDVI and *T_max_* for the vegetation growing season in 12.9% of the regions, none of them passed the significance test (*p* > 0.05) (Figure 4a).

When we removed the influence of *T_max_* and precipitation during the vegetation growing season, NDVI and *T_min_* were positively correlated in about 91.1% of the regions, among which 21.9% of the areas showed significant positive correlation (*p* < 0.05) and 27.9% of the regions showed very significant positive correlation (*p* < 0.01). Although about 8.9% of the study areas showed a negative correlation between NDVI and *T_min_*, none of them were significant (*p* > 0.05) (Figure 4b).

The correlation of NDVI and *T_min_* during the vegetation growing season that passed the significance test (*p* < 0.05) was 49.8% of the study areas, among which 56.0% was significant at the very significant level (*p* < 0.01). In the vegetation growing season, the correlation between NDVI and *T_max_* that passed the significance test (*p* < 0.05) was 43.6% of the Yellow River Basin, among which 46.1% very significantly passed the positive relationship test (*p* < 0.01). Based on the above analysis, we can conclude that vegetation in the Yellow River Basin has different response characteristics to the increasing daytime temperature. Vegetation NDVI has a more significant response to increasing night-time temperature, which indicates it has a wide impact on vegetation growth.

The areas where the partial correlation between NDVI and *T_max_* during the vegetation growing season passed the significance test are mainly distributed from Hekou town to Longmen, the eastern part of the inner flow area, and Longmen to Sanmenxia. The areas where the partial correlation between NDVI and *T_min_* during the vegetation growing season had passed the significance tests are mainly distributed from Hekou town to Longmen, from Longmen to Sanmenxia, and from Lanzhou to the lower part of Hekou town.

### 3.3. Partial Correlation between Different Vegetation NDVI and Daytime and Night-Time Warming

The study used partial correlation to distinguish the influence between *T_max_*, *T_min_* and NDVI of different types of vegetation in the growing season over the Yellow River Basin (Table 1). Except for grassland and meadow, coniferous forest, and broadleaf forest, the NDVI of cultivated plants, shrub, and desert is positively correlated with *T_max_*, which means the increasing *T_max_* during day has significant and positive impacts on cultivated plants, shrub, and desert vegetation growth, which could increase the vegetation coverage in the arid and semi-arid regions. The partial correlation between different types of vegetation and *T_min_* in the Yellow River Basin showed a common, very significant relationship (*p* < 0.01), and except for broadleaf forest, all the other vegetations are significantly positively related to the increasing *T*_min_. The increase of the *T_min_* has a more comprehensive and positive effect on vegetation.

## 4. Discussion

From 1982 to 2015, *T_max_* and *T_min_* in the vegetation growing season of the Yellow River Basin showed a significant increasing trend (*p* < 0.05), and the increasing rate of *T_min_* in the vegetation growing season was 1.25 times faster than that of *T_max_*. Previous studies have shown that the global surface temperature increased significantly faster at night than at day in the past 50 years, and the *T_min_* was 1.4 times higher than *T_max_* [7]. The general warming trend of the Yellow River Basin is consistent with the global trend. However, there are significant differences in the temperature changes between geographic locations and different time horizons, and six sub-basins (I, II, III, IV, V, VI, VII, and VIII) show different increasing trends in *T_max_* and *T_min_*_,_ i.e., a larger increase of *T_max_* accompanies a smaller rise in *T_min_*; while sub-basins of II and III have a similar increasing characteristic of both *T_max_* and *T_min_*. The driving mechanism under the pattern can be attributed to the influence of nature and human activities. Firstly, it has been reported that extreme temperature is closely related to elevation [47,48] and high altitude areas have faster increasing trend [49]. Therefore, climatic change trend varies across the Yellow River Basin due to its complex terrain. Meanwhile, the study area spanned 10° latitudes from north to south and three climate zones (arid, semi-arid, semi-humid) from southeast to northwest, which lead to different change trends across the study area. Secondly, the heat island effect causes differences in extreme temperature variations between urban stations and rural stations [50]. The effects of large-scale atmospheric circulation on extreme temperature changes, human greenhouse gas emissions [51], and changes in cloud cover [49] are also closely related to extreme temperatures. In addition, changes in land use and land cover by human activities could also cause the asymmetric warming [52]. The complex mechanism of action remains to be further studied in the future.

Depending on temperature as thermal energy, plants modulate the inner biogeochemical processes [53]. Therefore, rising *T_min_* is very important for vegetation growth and productivity, since it extends the length of growing season by increasing *T_min_*, and affects soil water and soil nitrogen processes. Most plants photosynthesize during daytime and are more sensitive to the maximum temperature, whereas plant respiration occurs throughout day and night, therefore influenced by both *T_max_* and *T_min_* [7]. So, the daytime and nighttime warming affects vegetation growth and production. The study found that, in general, daytime and night-time warming had a significant positive effect on NDVI in the vegetation growing season over the Yellow River Basin, 1982–2015. 

In most regions, *T_max_* and *T_min_* had promoting effects on NDVI, and in the rest places the day-night temperature increase had an inhibiting effect on NDVI. In the semi-humid regions, which have sufficient moisture, temperature becomes the main limiting factor to vegetation growth, and the increases of *T_max_* can promote the opening of stoma in vegetation leaves and vegetation transpiration, and therefore also increase the probability of carbon absorption and improve the availability of soil nitrogen. This promotes the vegetation growth, as indicated by increased NDVI [21]. The increasing of *T_max_* in the arid areas of the Yellow River Basin can accelerate leaf transpiration, leading to increased evaporation, reduced soil moisture, enhanced water stress, and ratarded growth and photosynthesis. This is consistent with the research results of Peng et al. [7], who found that an increase in daytime temperature was beneficial to vegetation growth and its ecosystem carbon sink function in most cold and humid regions, but not to the vegetation growth in temperate arid and semi-arid regions.

The vegetation during the growing season is dependent on and sensitive to temperature conditions and precipitation. The precipitation is being controlled for in the partial derivative analysis, but that does not mean the precipitation is not important and wouldn’t impact the observed NDVI trends. The annual precipitation in the upper reaches of the Yellow River Basin increased, and the change of precipitation is small in the middle and lower reaches (−1~1 mm/year) [54,55]. Meanwhile, the influence of precipitation change on vegetation has a great correlation with the climate zone. If the study area located in the humid zone, the slightly fluctuation of precipitation have little effect. Conversely, small fluctuations in precipitation will affect the growth of vegetation in the arid zone. For example, if precipitation significantly decreased while temperatures increased, then that could have a significant negative impact on vegetation growth, whereas if it increased that would generally have a positive impact on vegetation growth. Peng et al. [7] have shown that partial correlation between *T_max_* and NDVI during the growing season was positive in northwestern North America and Siberia and was negative in drier temperate regions (western China, central Eurasia, central and southwestern North America). The wet and dry regions of the Northern Hemisphere have opposite responses of NDVI to *T_min_*. For instance, an increase in *T_min_* can reduce rice yields by 10% per °C in the Philippines [56], but promotes growth in Inner Mongolia of China, where there is a temperate dry grassland site [20]. 

The mechanism of *T_max_* and *T_min_* on vegetation is different. The increase of *T_min_* causes the increase of plant respiration and the increase of nutrient metabolism rate in the body, resulting in increased loss of organic matter in plants [13,21], reducing the duration of the filling period and reducing the volume of endosperm cells, which could decrease vegetation productivity [8]. This may cause a negative partial correlation between vegetation NDVI and *T_min_*. On the other hand, the increase of *T_min_* may also reduce the frequency of frost disasters [57] and the compensatory effects of night-time autotrophic breathing [8] to increase aboveground biomass. This mechanism may lead to a positive partial correlation between certain vegetation NDVI and *T_min_*. It could also explain why broadleaf NDVI is not significantly related to either *T_max_* or *T_min_* and why most broadleaf vegetation in the Yellow River Basin are plantation forests, which are under extensive management and thus less sensitive to changes in both *T_max_* and *T_min_*.

The asymmetric temperature increase can affect the carbon absorption and carbon consumption of vegetation [58]. Shrubs in the Yellow River Basin show a positive correlation with temperature. Studies have found that the increase of deciduous shrubs is conducive to the increase of plant carbon reserves, because shrubs will distribute a large part of photosynthetic products to the stem, while the decomposition rate of the stem is slow [59].

## 5. Conclusions

This study analyzes the spatial distribution of maximum temperature (*T_max_*) and the minimum temperature (*T_min_*) over the Yellow River Basin and the relationship between vegetation NDVI and maximum temperature (*T_max_*) and the minimum temperature (*T_min_*), using GIMMS-NDVI3g data and meteorological data in the growing season from 1982 to 2015. The results provide a primary foundation for the study of diurnal asymmetry and its impacts on diverse vegetations across a large basin. The Yellow River Basin has experienced a trend of asymmetric warming, and the minimum temperature has increased faster than maximum temperature from 1982 to 2015. The rate of increase of *T_min_* was 1.25 times faster than that of *T_max_*. The warming trend and magnitude of *T_min_* and *T_max_* across the Yellow River Basin are also spatially heterogeneous.

According to the best of our knowledge, this study is the first to find that in the Yellow River Basin, cultivated plants, shrubs, and deserts are significantly and positively related to both the increasing maximum temperature and minimum temperature. This finding may indicate that crop yields, shrub growth, and desert vegetation in the Yellow River Basin are experiencing enhanced growth caused by the increasing *T_max_* and *T_min_*. However, crop yields are particularly sensitive to anthropogenic factors such as improved farming infrastructure, changing crop types, and genetic engineering. The shrub growth and desert vegetation may be affected by anthropogenic factors such as fire, felling and green projects. The significant increases of the minimum temperature of the Yellow River Basin have a very positive effect on all vegetation types except broadleaf forests. Vegetation NDVI in the Yellow River Basin has different responses to diurnal warming with more significant responses to *T_min_*, which affects most vegetation types across the Yellow River Basin. Meanwhile, different vegetations types have different responses to asymmetric warming. Daytime warming (i.e., the maximum temperature increase) had negative influences on grassland and meadow, coniferous forests and broadleaf forests. However, daytime warming had positive influences on cultivated plants, shrubs and desert, which means that the increasing maximum temperature could improve crop yield and increase vegetation growth across the arid and semi-arid areas in the Yellow River Basin.

The spatio-temporal variation of vegetation NDVI is the result of a combination of climate change and human activity. The correlation between maximum temperature (*T_max_*) and the minimum temperature (*T_min_*), and vegetation NDVI is analyzed in our study, but nutrient availability, solar radiation, and the anthropogenic factors should be taken into account in further research. In addition, precipitation is also important for vegetation growing and would impact the NDVI trends under asymmetric warming. We will research the impacts of change precipitation on vegetation growing under asymmetric warming in the future work to expound the influence of complex climate change. Meanwhile, our studies used 8 × 8 km mixed pixel NDVI data, which typically reflects the mixed spectrum from several land cover/land use types. The next step study would combine other higher resolution image data, such as MODIS NDVI, and MODIS Nadir BRDF-adjusted(Bidirectional Reflectance Distribution Function)surface reflectance, using data-fusion to impute the 1982–2000 images to achieve 250 m remote-sensing images as the MODIS NDVI data from 2000–2015 to reduce the influence of pixel mixed effect. Due to the longest period in our study, the land use/land cover inevitably changed. In the future, we will eliminate quantitatively the dynamics in vegetation caused by land cover changes in the case of obtaining yearly land use change map. The asymmetric warming effects can be further quantified by combining the observation experimental data from ground stations and model simulation analyses to provide a more reliable scientific basis for ecological environment monitoring and adaptation to climate change. Vegetation NDVI is a comprehensive response to climate parameters, and future research may consider the effects of other meteorological factors such as humidity, evaporation, and sunshine time on vegetation NDVI.

## Figures and Tables

**Table 1:** Partial correlation coefficients between NDVI and *T_max_*/*T_min_* for different vegetation types in the Yellow River over the period 1982–2015.

Vegetation Types	*T_max_*	*T_min_*	Area (km^2^)
coniferous forest	0.142^NS^	0.567**	304
cultivated plants	0.599**	0.528**	2864
broadleaf forest	0.289^NS^	–0.217^NS^	402
shrub	0.557**	0.657**	686
desert	0.418*	0.537**	397
Grassland and meadow	–0.307^NS^	0.661**	3434

** *p* < 0.01,* *p* < 0.05, *p* > 0.05 NS (non-significant).
